# Supplementary material for: Psychometric validation of the Thai version of the 12-item Zarit Burden Interview among informal caregivers in a palliative care setting in the south of Thailand
Source: PLoS One. 2025 May 15;20(5):e0322852. doi: 10.1371/journal.pone.0322852 (PMC12080790; doi:10.1371/journal.pone.0322852)
Supplement: Attachment — Submitted filename: Opinion_Validation_of_the_Thai version_ZBI 12.pdf [file pone.0322852.s002.pdf]

Dear,

The manuscript titled **“Validation of the Thai Version of the 12-Item Zarit Burden Interview Among Informal Caregivers in a Palliative Care Setting”** has been evaluated. The objective of the study was to validate the psychometric properties of the 12-item ZBI in Thai caregivers of palliative patients.

Based on my evaluation, I recommend that the manuscript be **accepted with major revisions.** While the study addresses an important and impactful topic, some areas require improvement. Below are my suggestions for the manuscript. I would also like to take this opportunity to thank you for allowing me to review this work.

1) Title: I suggest that the authors modify the title to "Psychometric Validation of the Thai Version of the 12-Item Zarit Burden Interview Among Informal [...]" to improve clarity and precision.

Abstract and Key-words: Both are suitable.

2) Introduction:

- I suggest that the acronym “WHO” be written out in full the first time it appears, with WHO in parentheses.
- In the phrase “According to this description...”, I recommend that the authors include at least one reference to support this statement.
- If the Zarit Burden Interview (ZBI) is one of the most widely used self-report questionnaires, I suggest including additional references to substantiate this claim.
- I also recommend that the justification for this study be more detailed. Since self-report questionnaires offer several advantages in clinical settings—such as low implementation costs and shorter administration times—highlighting these aspects could strengthen the rationale for the study.
- Include justifications in the text for why informal caregivers were chosen as the sample.
- In line 131, I suggest that the authors specify the good psychometric properties of the DASS-21 Thailand and explain why this scale was chosen over another.

3) Methods:

Study Design and Population:

- I suggest that the inclusion and exclusion criteria be explicitly stated to ensure clarity.
- What theoretical background was used to determine the criteria of three months and three days per week? I recommend providing a reference to support this choice.
- Since the sample includes both sexes, I suggest explicitly mentioning this in the inclusion criteria.
- How were the participants randomly selected? This process needs to be described in detail.
- In line 131, I suggest that the authors specify the good psychometric properties of the DASS-21 Thailand and explain why this scale was chosen over another.

4) Strengths and Limitations:

- The results are well described and effectively represented in tables and figures. I suggest that the authors check the quality of Figure 1, as it appears blurry.

5) Strengths and Limitations:

- The limitations presented by the authors are valid and encourage reflections on how future studies can build upon these findings.

6) Conclusion:

- I suggest reformulating the conclusion, as some sentences included in it would be more appropriate in the limitations section.

I suggest that the authors review the manuscript's English.
