# Supplementary material for: Psychometric validation of the Thai version of the 12-item Zarit Burden Interview among informal caregivers in a palliative care setting in the south of Thailand
Source: PLoS One. 2025 May 15;20(5):e0322852. doi: 10.1371/journal.pone.0322852 (PMC12080790; doi:10.1371/journal.pone.0322852)
Supplement: Attachment — Submitted filename: Response to Reviewers.docx [file pone.0322852.s004.docx]

**Responses to reviewers on MS version dated 3 March 2025**

Psychometric Validation of the Thai version of the 12-item Zarit Burden Interview among informal caregivers in a palliative care setting

***Journal Requirements***

| ***Journal Requirements*** | ***Response from author*** |
| --- | --- |
| 1) Please ensure that your manuscript meets PLOS ONE's style requirements, including those for file naming. | ***Response 1:***  Thank you for your suggestion for improving our manuscript. We have reviewed the PLOS ONE's style requirements and ensured that our manuscript complies with the specified formatting, including file naming conventions.  Please see the revised manuscript. |
| 2) Please amend your list of authors on the manuscript to ensure that each author is linked to an affiliation. Authors’ affiliations should reflect the institution where the work was done (if authors moved subsequently, you can also list the new affiliation stating “current affiliation:….” as necessary). | Thank you for your comment regarding the authors' affiliations. We have ensured that the listed affiliations accurately reflect the institutions where the research was conducted. Also, our research team consists of members from both academic institutions and healthcare service settings, in alignment with our concept of the academic-practice approach. This interdisciplinary collaboration was essential in strengthening our research team. |

**Response to Editor:**

| ***Comments*** | ***Response from author*** |
| --- | --- |
| 1) Title: I suggest that the authors modify the title to "Psychometric Validation of the Thai Version of the 12-Item Zarit Burden Interview Among Informal [...]" to improve clarity and precision. | ***Response 1:***  Thank you for your suggestion for improving our manuscript. We modified the title of our manuscript and followed your suggestion.  Please see the revised manuscript. |
| 2) Introduction:  I suggest that the acronym “WHO” be written out in full the first time it appears, with WHO in parentheses. | ***Response2:***  Thank you for your suggestion. We added the acronym “WHO” in our manuscript.  (in line 49)  Please see the revised manuscript. |
| In the phrase “According to this description...”, I recommend that the authors include at least one reference to support this statement. | ***Response3:***  Thank you for your suggestion. We include one reference to support this statement following your suggestion. (in line 53)  Radbruch L, De Lima L, Knaul F, Wenk R, Ali Z, Bhatnaghar S, et al. Redefining palliative care—a new consensus-based definition. Journal of pain and symptom management. 2020;60(4):754-64. https:// doi: 10.1016/j.jpainsymman.2020.04.027. PMID: 32387576  Please see the revised manuscript |
| If the Zarit Burden Interview (ZBI) is one of the most widely used self-report questionnaires, I suggest including additional references to substantiate this claim | ***Response4:***  Thank you for your suggestion. As the Zarit Burden Interview (ZBI) is one of the most widely used self-report questionnaires, we include additional references to substantiate this claim. (in line 76)  Please see the revised manuscript |
| I also recommend that the justification for this study be more detailed. Since self-report questionnaires offer several advantages in clinical settings—such as low implementation costs and shorter administration times—highlighting these aspects could strengthen the rationale for the study. | ***Response5:***  Thank you for your suggestion. We added this statement to our manuscript to support several advantages of self-report questionnaires.  These self-report questionnaires provide various advantages in clinical settings, including minimal implementation costs and shorter administration times. (in line 78-79)  Please see the revised manuscript |
| Include justifications in the text for why informal caregivers were chosen as the sample. | ***Response6:***  Thank you for your suggestion. We included the statement of informal caregiver to clarify its definition and support our justification.  There are 2 types of caregivers formal and informal. The formal caregiver is the individual who is compensated for the service. The informal caregiver is typically a family member, friend, or neighbor. Although this type of caregiver is neither paid nor trained to conduct the function, they have a great degree of dedication as evidenced by their affective relationship [4]. In the case of palliative patients, the type of care in healthcare is usually informal, with the family member being the primary caregiver. This situation has impacted numerous caregivers of individuals with advanced or terminal illnesses, leading to a significant burden [5]. (in line 57-63)  Please see the revised manuscript |
| In line 131, I suggest that the authors specify the good psychometric properties of the DASS-21 Thailand and explain why this scale was chosen over another. | ***Response7:***  Thank you for your suggestion. We add the reliability of the Thai version of DASS-21 to support our justification.  The Cronbach’s alpha coefficient for the Thai version of the DASS-21 was 0.92.  (in line 141-142)  Please see the revised manuscript |
| 3) Methods:  Study Design and Population:  I suggest that the inclusion and exclusion criteria be explicitly stated to ensure clarity | ***Response8:***  Thank you for your suggestion. We added the inclusion and exclusion criteria for clarifying the enrolled sample in this study.  The inclusion criteria for this study were the primary caregivers who included friends, family members, and individuals involved in PC, through patients receiving treatment in medical institutions or at home through two district health service networks. They were male or female Thai nationals, aged 18 or above, who had provided care for a minimum of 3 days per week for at least 3 months without payment [24]. The exclusion criteria were caregivers with poor general conditions or having mental disorders such as schizophrenia, psychosis, or dementia. (in line 117-122)  Please see the revised manuscript |
| What theoretical background was used to determine the criteria of three months and three days per week? I recommend providing a reference to support this choice. | ***Response9:***  Thank you for your suggestion. However, the duration of care varies across previous studies, depending on the condition of the patients. We attempted to provide a reference to support this statement (see line 120).  Koopman E, Heemskerk M, van der Beek AJ, Coenen P. Factors associated with caregiver burden among adult (19–64 years) informal caregivers–An analysis from Dutch Municipal Health Service data. Health & social care in the community. 2020;28(5):1578-89.  https://doi: 10.1111/hsc.12982. PMID: 32207221  Please see the revised manuscript |
| Since the sample includes both sexes, I suggest explicitly mentioning this in the inclusion criteria. | ***Response10:***  Thank you for your suggestion. We added this statement to our inclusion criteria.  They were male or female Thai nationals, aged 18 or above, who had provided care for a minimum of 3 days per week for at least 3 months without payment.  (in line 119-120)  Please see the revised manuscript |
| How were the participants randomly selected? This process needs to be described in detail | ***Response10:***  Thank you for your comments. The participants enrolled in this study did not randomly select as they came from the health service network that we mentioned in Study design and population. However, we included additional details to clarify this statement.  The family caregivers of palliative patients served by the hospital support team and the home care team were recruited consecutively. (in line 115-116)  Please see the revised manuscript |
| In line 131, I suggest that the authors specify the good psychometric properties of  the DASS-21 Thailand and explain why this scale was chosen over another. | ***Response11:***  Thank you for your suggestion. We add the reliability of the Thai version of DASS-21 to support our justification.  The Cronbach’s alpha coefficient for the Thai version of the DASS-21 was 0.92.  (in line 141-142)  Please see the revised manuscript |
| 4) Strengths and Limitations:  The results are well described and effectively represented in tables and figures. I suggest that the authors check the quality of Figure 1, as it appears blurry | ***Response12:***  Thank you for your suggestion. We edit our figure following the journal recommendations. (in line 241-243)  Please see the revised manuscript |
| 5) Strengths and Limitations:  The limitations presented by the authors are valid and encourage reflections on how future studies can build upon these findings. | ***Response13:***  Thank you for your opinion. We appreciated your comments and attempt to improve our manuscript following your suggestion. |
| 6) Conclusion:  • I suggest reformulating the conclusion, as some sentences included in it would be  more appropriate in the limitations section. | ***Response14:***  Thank you for your suggestion. We reformulate some parts of our conclusion that would be the imitation.  This study offers psychometric validation for the 12-item ZBI as a reliable tool to assess the burden of care experienced by palliative caregivers. We have gathered compelling evidence to support reliability, convergent validity, and construct validity in this population. Factor analysis confirms a two-dimensional internal structure. As a result, this version of the ZBI enables a more efficient and useful burden assessment and health care providers can utilize this tool in both research and clinical environments to evaluate the level of burden experienced by palliative caregivers.  (in line 355-361)  Please see the revised manuscript |
| I suggest that the authors review the manuscript's English. | ***Response15:***  Thank you for your suggestion. We reviewed the grammatical of our manuscript and he accuracy of English language, grammar, punctuation, spelling and overall style were edit and proof by native from International College for Sustainability Studies. We also sent the file of certification of language proof via the first submission. |

**Response to reviewer 1**

| ***Reviewer 1 comments*** | ***Response from author*** |
| --- | --- |
| Methods:  - Lines 108-112: If the authors included friends as caregivers, they should avoid calling them “family caregivers”. Informal (non-paid) caregivers seem like a better term if friends are also included. | ***Response 1:***  Thank you for your suggestion for improving our manuscript. After we reviewed the definition of informal caregivers following your suggestion we agree with your comments and edited this term to “informal caregivers”  (In line 115)  Please see the revised manuscript. |
| Lines 112-113: Do the authors know the minimum hours per day that were provided each day? | ***Response 2:***  Thank you for your comments. Please accept our apology for our error, however we reviewed and revised these minimum hours of caring per day and added the reference for clarifying this definition.  They were male or female Thai nationals, aged 18 or above, who had provided care for a minimum of 3 days per week for at least 3 months without payment [24].  (In line 119-121)  Please see the revised manuscript. |
| Lines 122-123: Is the 12-item Zarit Burden Interview scale validated in Thai? Or just the 22-item version? This is very important. If it is not validated, it should be mentioned in the limitations section. | ***Response 3:***  Thank you for your comments. The Thai version of 12-item ZBI has been validated in caregiver of psychiatric patients.    The Thai version of the 12-item ZBI (ZBI-12) has been validated in of psychiatric patients with an excellent internal consistency [12].  (In line 128-129)  Please see the revised manuscript. |
| Lines 138-140: Please detail the randomization process. How was it performed? | ***Response 4:***  Thank you for your comments. The participants enrolled in this study did not randomly select as they came from the health service network that we mentioned in Study design and population. However, we included additional details to clarify this statement.  The family caregivers of palliative patients served by the hospital support team and the home care team were recruited consecutively. (in line 115-116)  Please see the revised manuscript |
| Line 144: Please change to “All statistical analyses in this study were conducted using…” | ***Response 5:***  Thank you for your suggestion for improving our manuscript.  We edited this statement following your suggestion. (in line 154)  Please see the revised manuscript |
| Results:  - I have several comments for Table 1:  o Please specify the “other relative”. How many of those were friends or non-family caregivers? | ***Response 6:***  Thank you for your suggestion for improving our results.  We added the details of other relatives (friends, neighbors) in table 1  Please see the revised manuscript |
| What do you mean by caregiver with illness? What type of illness? There is a difference between having dyslipidemia or having rheumatoid arthritis, for example. | ***Response 7:***  Thank you for your comments about improving our results.  Please accept our apology as we did not collect the details of caregiver health problems in our questionnaire. The question for information details is whether the caregivers have any health problems or not. Thanks for your suggestion. |
| Family members living with the caregiver or with the patient? | ***Response 8:***  Thank you for your comments about improving our results.  We added this detail with “Family members living with the patient”  Please see the revised manuscript |
| Providing care between 1 and 10 hours per week (22%) seems too little to me. If one of the inclusion criteria includes providing care at least three days a week, how is it possible to provide 1 hour of care? This is a bit confusing. I can’t believe someone provides only 20 minutes per care a day. | ***Response 9:***  Thank you for your comments. Please accept our apology for our error as we collected data for both the number of day care /week and care hour/day and confused for using data analyses in our previous manuscript as you think someone provides only 20 minutes per care a day. (We are confused about the number of day care and number of hour care/day). However, we reviewed and revised this minimum care hours/ day instead of the wording existing in our previous day of care hour/week, and also added the reference for clarifying this definition.  They were male or female Thai nationals, aged 18 or above, who had provided care for a minimum of 3 days per week for at least 3 months without payment [24].  (In line 119-121). In addition, we reviewed and revised our analysis and edited the number of hours followed by both the definition and the number of care hour/day instead of the number care hour/week that existing in the previous manuscript.  Please see the revised manuscript |
| The limitations section would benefit from including all the actual limitations of the study. Most of your caregivers have been providing care for a short time (<5 years) and for a very short number of hours per week (<10 hours, 22%). Are you using a 12-item Thai validated ZBI? Or just the 22-item ZBI has been validated? | ***Response 10:***  Thank you for your suggestion. After we reviewed our data and revised our analysis, we found some mistakes and accepted your comments. We carefully checked and edited these statements to clarify these results. We edited care hour/week to care hour/day following our data analysis.  Please see the revised manuscript |

**Response to reviewer 2**

| ***Reviewer 2 comments*** | ***Response from author*** |
| --- | --- |
| Limitations:  This validation study of the ZBI-12 can contribute to clinical care, both in outpatient and hospital settings, as well as to scientific research, which can benefit from this instrument. I suggest that the authors review the English language. | ***Response 9:***  Thank you for your suggestion.  We edited these statements following your suggestion in the conclusion of this study.  (In line 358-362).  Please see the revised manuscript |

I would like to express my gratitude for your diligent efforts in providing comments and suggestions to improve our manuscript. We sincerely appreciate your feedback and have revised the manuscript accordingly. If you have any further comments, we are happy to make additional edits and resubmit the manuscript as quickly as possible.

We have corrected the data in Table 1, as we initially confused the number of care hours per week with the number of care hours per day. We sincerely apologize for this mistake. We confirm that the values presented in Table 1 now accurately reflect care hours per day.

Finally, we appreciate your comments and have carefully reviewed and revised our dataset, ensuring the correct representation of care hours.
