# Supplementary material for: Psychometric validation of the Thai version of the 12-item Zarit Burden Interview among informal caregivers in a palliative care setting in the south of Thailand
Source: PLoS One. 2025 May 15;20(5):e0322852. doi: 10.1371/journal.pone.0322852 (PMC12080790; doi:10.1371/journal.pone.0322852)
Supplement: Attachment — Submitted filename: Response_to_Reviewers_auresp_2.docx [file pone.0322852.s005.docx]

**Responses to reviewers on MS version dated 20 March 2025**

Psychometric validation of the Thai version of the 12-item Zarit Burden Interview among informal caregivers in a palliative care setting in the south of Thailand.

***Journal Requirements***

| ***Journal Requirements*** | ***Response from author*** |
| --- | --- |
| 1) Please review your reference list to ensure that it is complete and correct. If you have cited papers that have been retracted, please include the rationale for doing so in the manuscript text or remove these references and replace them with relevant current references. Any changes to the reference list should be mentioned in the rebuttal letter that accompanies your revised manuscript. If you need to cite a retracted article, indicate the article’s retracted status in the References list and also include a citation and full reference for the retraction notice. | ***Response 1:***  Thank you for your suggestion for improving our manuscript. We reviewed all references in this manuscript and no papers in our manuscript that have been retracted.  In addition, we added 2 new references following the reviewer’s suggestion in reference order no24 and order no.37., so the reference list has been changed as follows:  24. Mbuagbaw L, Lawson DO, Puljak L, Allison DB, Thabane L. A tutorial on methodological studies: the what, when, how and why. BMC Medical Research Methodology. 2020;20:1-12. https://doi.org/10.1186/s12874-020-01107-7. PMID: 32894052.  37. Whitlatch CJ, Zarit SH, von Eye A. Efficacy of interventions with caregivers: a reanalysis. The Gerontologist. 1991;31(1):9-14. https://doi.org/ 10.1093/geront/31.1.9. PMID: 2007480.  Please see the revised manuscript. |

**Response to Reviewer 2:**

| ***Comments*** | ***Response from author*** |
| --- | --- |
| I would like to thank you for making all the necessary changes and accepting the suggestions in this report. I congratulate the authors on their excellent work, which will certainly add value to scientific literature. I hope this manuscript will be available to the community soon | ***Response 1:***  Thank you for all your suggestions to help us improve our manuscript. |

**Response to reviewer 3**

| ***Reviewer 3 comments*** | ***Response from author*** |
| --- | --- |
| 1) Regarding the study’s limitations, it is necessary to consider:  - The study employed a cross-sectional design, meaning that data were collected at a single point in time. This design does not allow for the assessment of test-retest reliability or the stability of measurements over time. Consequently, it limits the ability to draw conclusions about the long-term applicability of the 12-item Zarit Burden Interview (ZBI) in measuring caregiver burden. | ***Response 1:***  Thank you for your suggestion for improving our manuscript. We agreed with your opinion and added these statements in both part of the limitation and the conclusion of our study.  Limitation:  Moreover, this study employed a methodological study in which data was collected at a single point in time. It does not allow for the evaluation of test-retest reliability or long-term measurement stability. As a result, it limits the capacity to draw conclusions regarding the 12-item ZBI's long-term usefulness in measuring caregiver burden. (In line 356-359)  Conclusions:  Furthermore, the additional validation studies of the 12-item ZBI using longitudinal design are warranted to ensure its long-term measurement stability.  (In line 368-369)  Please see the revised manuscript. |
| The confirmatory factor analysis indicated that negative emotions were primarily linked to caregivers’ depressive symptoms. This suggests that the adverse experiences of caregiving may have a more significant impact on mental well-being than the distress experienced by care recipients. This focus may overlook other important dimensions of caregiver burden. | ***Response 2:***  Thank you for your comments and suggestions. After we reviewed previous studies, our results are comparable to other studies that aimed to validate the 12-item Zarit Burden Interview; however, some studies reported another dimension, such as “guilt.” It remains possible that a three-factor structure is unique to the 22-item ZBI, but it accounts for the lack of a third factor found with the 12-item ZBI in this study and previous studies. As a result, please allow us to add your additional details to our discussion part.  However, this does not imply that other dimensions of caregiver burden are nonexistent. This highlights the necessity for further research on other factors that may contribute to increasing stress among caregivers. (In line 337-340)  Please see the revised manuscript. |
| 2) Other comments:  - The study is better classified as a methodological study, although some publications refer to it as a “cross-sectional study.” I suggest reviewing this classification based on the available literature. | ***Response 3:***  Thank you for your suggestion. We reviewed and edited our study design, followed your suggestion, and added the reference to support this classification.  This methodological study focused on the validity of the 12-item ZBI, utilizing data from the "palliative care outcomes and factors associated with the quality of life of caregivers of palliative care patients" study [24]., (In line 109-111)  24. Mbuagbaw L, Lawson DO, Puljak L, Allison DB, Thabane L. A tutorial on methodological studies: the what, when, how and why. BMC Medical Research Methodology. 2020; 20:1-12.  https://doi.org/10.1186/s12874-020-01107-7. PMID: 32894052.  Please see the revised manuscript |
| There are minor grammar and typographical errors, such as "circumstance" in the abstract, which should be "context." I suggest reviewing the grammar and spelling of the text. | ***Response 4:***  Thank you for your suggestion for improving our manuscript.  We edited this statement following your suggestion, and we carefully reviewed the grammar and spelling of the text in this manuscript.  (In line 24)  Please see the revised manuscript |
| - The justification for the division into "personal strain" and "role strain" could be better supported theoretically. | ***Response 5:***  Thank you for your suggestion for improving our results.  We added the details and definitions of the 2 factors, namely "personal strain" and "role strain," that existed in the original version of the 12-item ZBI.  These two factors are based on the factor structure of the ZBI-22 described by Whitlatch [37]. Typically, "personal strain" refers to caregivers' subjective and emotional reactions to many aspects of caregiving. The term "role strain" refers to the practical aspects of caregiving, rather than an individual's personal experience. Indeed, it can be adequate. described as "the stress due to role conflict or overload" [37].  (In line 281-285)  37. Whitlatch CJ, Zarit SH, von Eye A. Efficacy of interventions with caregivers: a reanalysis. The Gerontologist. 1991;31(1):9-14. https://doi.org/ 10.1093/geront/31.1.9. PMID: 2007480.  Please see the revised manuscript |
| - The layout of the tables should be revised, or they should be labeled as "figures." Additionally, the titles should include the period and location (country) where the study was conducted. | ***Response 6:***  Thank you for your comments about improving our results.  We present our CFA with figure 1. Regarding the layout of the tables, after careful consideration, we have decided to maintain their current format, as they effectively convey the data in a clear and concise manner.  We included the setting of our study in the titles.  Psychometric validation of the Thai version of the 12-item Zarit Burden Interview among informal caregivers in a palliative care setting in the south of Thailand.  Please see the revised manuscript |
| - The conclusion should be revised in light of the considerations/comments presented. | ***Response 7:***  Thank you for your comments about improving our conclusion.  Furthermore, the additional validation studies of the 12-item ZBI using longitudinal design are warranted to ensure its long-term measurement stability.  (In line 368-369)  Please see the revised manuscript |

Finally, we would like to express our sincere gratitude to the reviewers for their thoughtful and constructive feedback on our manuscript. Their insightful comments have been invaluable in refining our work and enhancing its clarity and quality. We appreciate any additional feedback you may have and are ready to make further revisions. We will resubmit the manuscript promptly after addressing your comments.
